# Supplementary material for: OpenMM 7: Rapid development of high performance algorithms for molecular dynamics
Source: PLoS Comput Biol. 2017 Jul 26;13(7):e1005659. doi: 10.1371/journal.pcbi.1005659 (PMC5549999; doi:10.1371/journal.pcbi.1005659)
Supplement: S1 Source Code — (ZIP) [file pcbi.1005659.s002.zip › OpenMM-7.0.1-Source/docs/api-c++/generated/OpenMM.State.html]

State — OpenMM @OPENMM\_MAJOR\_VERSION@.@OPENMM\_MINOR\_VERSION@ documentation


# State¶

*class* `OpenMM::``State`¶
:   A `State` object records a snapshot of the current state of a simulation at a point in time. You create it by calling getState() on a `Context`.

    When a `State` is created, you specify what information should be stored in it. This saves time and memory by only copying in the information that you actually want. This is especially important for forces and energies, since they may need to be calculated. If you query a `State` object for a piece of information which is not available (because it was not requested when the `State` was created), it will throw an exception.

    Internal class used to construct new `State` objects.

    Methods

    |  |  |
    | --- | --- |
    | `State` | Construct an empty `State` containing no data. |
    | `getTime` | Get the time for which this `State` was created. |
    | `getPositions` | Get the position of each particle. |
    | `getVelocities` | Get the velocity of each particle. |
    | `getForces` | Get the force acting on each particle. |
    | `getKineticEnergy` | Get the total kinetic energy of the system. |
    | `getPotentialEnergy` | Get the total potential energy of the system. |
    | `getPeriodicBoxVectors` | Get the vectors defining the axes of the periodic box (measured in nm). |
    | `getPeriodicBoxVolume` | Get the volume of the periodic box (measured in nm^3). |
    | `getParameters` | Get a map containing the values of all parameters. |
    | `getDataTypes` | Get which data types are stored in this `State`. |
    | `StateBuilder` |  |
    | `getState` |  |
    | `setPositions` |  |
    | `setVelocities` |  |
    | `setForces` |  |
    | `setParameters` |  |
    | `setEnergy` |  |
    | `setPeriodicBoxVectors` |  |

    Enum: DataType

    |  |  |
    | --- | --- |
    | **Positions** |  |
    | **Velocities** |  |
    | **Forces** |  |
    | **Energy** |  |
    | **Parameters** |  |

    `State`()¶
    :   Construct an empty `State` containing no data. This exists so `State` objects can be used in STL containers.

    double `getTime`() *const*¶
    :   Get the time for which this `State` was created.

    *const* std::vector<Vec3> &`getPositions`() *const*¶
    :   Get the position of each particle. If this `State` does not contain positions, this will throw an exception.

    *const* std::vector<Vec3> &`getVelocities`() *const*¶
    :   Get the velocity of each particle. If this `State` does not contain velocities, this will throw an exception.

    *const* std::vector<Vec3> &`getForces`() *const*¶
    :   Get the force acting on each particle. If this `State` does not contain forces, this will throw an exception.

    double `getKineticEnergy`() *const*¶
    :   Get the total kinetic energy of the system. If this `State` does not contain energies, this will throw an exception.

        Note that this may be different from simply mv:cpp:any:Integrator is being used.

    double `getPotentialEnergy`() *const*¶
    :   Get the total potential energy of the system. If this `State` does not contain energies, this will throw an exception.

    void `getPeriodicBoxVectors`(Vec3 &*a*, Vec3 &*b*, Vec3 &*c*) *const*¶
    :   Get the vectors defining the axes of the periodic box (measured in nm).

        |  |  |
        | --- | --- |
        | Parameters: |  |

        - **a** – [out] the vector defining the first edge of the periodic box
        - **b** – [out] the vector defining the second edge of the periodic box
        - **c** – [out] the vector defining the third edge of the periodic box

    double `getPeriodicBoxVolume`() *const*¶
    :   Get the volume of the periodic box (measured in nm^3).

    *const* std::map<std::string, double> &`getParameters`() *const*¶
    :   Get a map containing the values of all parameters. If this `State` does not contain parameters, this will throw an exception.

    int `getDataTypes`() *const*¶
    :   Get which data types are stored in this `State`. The return value is a sum of DataType flags.

    `StateBuilder`(double *time*)¶

    State `getState`()¶

    void `setPositions`(*const* std::vector<Vec3> &*pos*)¶

    void `setVelocities`(*const* std::vector<Vec3> &*vel*)¶

    void `setForces`(*const* std::vector<Vec3> &*force*)¶

    void `setParameters`(*const* std::map<std::string, double> &*params*)¶

    void `setEnergy`(double *ke*, double *pe*)¶

    void `setPeriodicBoxVectors`(*const* Vec3 &*a*, *const* Vec3 &*b*, *const* Vec3 &*c*)¶

High performance molecular simulation on GPUs


### Navigation

- Core Objects
- Forces
- Integrators
- Extras

©2015, Stanford University and the Authors.
|
Powered by Sphinx 1.3.1
& Alabaster 0.7.7
|
Page source
